# Ab Initio Theory of Photoemission from Graphene

**DOI:** 10.3390/nano11051212

**Published:** 2021-05-03

**Authors:** Eugene Krasovskii

**Affiliations:** Departamento de Polímeros y Materiales Avanzados: Física, Química y Tecnología, Universidad del Pais Vasco/Euskal Herriko Unibertsitatea, 20080 Donostia/San Sebastián, Basque Country, Spain; eugene.krasovskii@ehu.eus

**Keywords:** graphene, angle-resolved photoemission, electron scattering, augmented plane waves

## Abstract

Angle-resolved photoemission from monolayer and bilayer graphene is studied based on an ab initio one-step theory. The outgoing photoelectron is represented by the time-reversed low energy electron diffraction (LEED) state $\Phi_{\mathrm{LEED}}^{*}$, which is calculated using a scattering theory formulated in terms of augmented plane waves. A strong enhancement of the emission intensity is found to occur around the scattering resonances. The effect of the photoelectron scattering by the underlying substrate on the polarization dependence of the photocurrent is discussed. The constant initial state spectra $I(k_{\left|\right|},\hbar \omega )$ are compared to electron transmission spectra T(E) of graphene, and the spatial structure of the outgoing waves is analyzed. It turns out that the emission intensity variations do not correlate with the structure of the T(E) spectra and are caused by rather subtle interference effects. Earlier experimental observations of the photon energy and polarization dependence of the emission intensity $I(k_{\left|\right|},\hbar \omega )$ are well reproduced within the dipole approximation, and the Kohn–Sham eigenstates are found to provide a quite reasonable description of the photoemission final states.

## 1. Introduction

Owing to the combination of high structural stability and unique electronic properties [1], graphene has become a paradigm two-dimensional material and a subject of numerous experimental and theoretical studies. The majority of research has addressed the vicinity of the Dirac point (DP), however, also the unbound states were discovered to exhibit fascinating phenomena, such as the electron-transmission slits at low kinetic energies [2,3,4] caused by the interlayer scattering and the scattering resonances due to the coupling of the in-plane and perpendicular motions at higher energies [5,6,7]. A detailed knowledge of the properties of unbound states is important for the interpretation of angle-resolved photoemission (ARPES), which is the most direct source of information about the occupied states. Graphene has been extensively studied with ARPES [8,9,10,11,12,13,14,15,16,17,18,19], and apparent final state effects were reported [9,13,14,18,19]. In particular, the circular dichroism [12,14,17] is of special interest owing to its close relation to the topological character of 2D states [20].

A characteristic feature of photoemission from graphite [21,22] and graphene [10,13,18] is the so-called “dark corridor”, i.e., the suppression of emission with the *p*-polarized light from the occupied π states along the $\bar{\Gamma }\bar{K}$ line in the second Brillouin zone (BZ) as a result of a destructive interference of the contributions to the photoemission matrix element from the two equivalent sublattices. In the monolayer graphene, the suppressed initial states are odd on reflection in the $\bar{\Gamma }\bar{K}$ line [18,23], so the dark corridor can be illuminated by the *s*-polarized light incident along $\bar{\Gamma }\bar{K}$ [13]. In Ref. [13] this was demonstrated experimentally, and, in addition, a strong photon-energy dependence of the emission intensity was revealed. These observations were analyzed using a multiple-scattering implementation [24] of the one-step theory of photoemission [25,26,27,28,29] based on a density-functional-derived one-particle potential. However, for a direct comparison between experiment and theory, the authors shifted the theoretical photon energies by 8.6 eV toward higher photon energies. The origin of such a large shift is unclear, especially in view of the fact that the LEED theory based on Kohn–Sham states describes the unoccupied continuum of both bulk graphite [30] and graphene [5,6,31,32,33] rather accurately. However, in Ref. [13] only two photon energies were studied, which may be insufficient for a conclusive comparison between experiment and theory. A more detailed measurement in the range ℏω=42–55 eV was reported in Ref. [18], and a rapid variation of the relative intensity for *s* and *p* light polarizations was observed.

A consistent and rigorous approach to photoemission is offered by the one-step theory [25,26,27,28,29], in which the photoexcitation and photoelectron transport to the detector (including elastic and inelastic scattering) are described by the time reversed LEED state $\Phi_{\mathrm{LEED}}^{*}$. Here, the one-step theory is applied to the monolayer and bilayer graphene with the aim to explain the experimentally observed features and analyze their relation to the properties of the relevant scattered waves. The final-state wave function $\Phi_{\mathrm{LEED}}^{*}$ is calculated using the augmented plane waves scattering formalism [34]. The present theory reproduces well both experiments [13,18] and reveals rapid variations of the character of the outgoing photoelectron wave with energy. These variations manifest themselves also in the electron transmission through the films and in the variations of the dwell time, i.e., the probability to find the scattered electron inside the film. However, the variations of these quantities do not correlate with each other, so the full knowledge of the wave function is necessary to describe the experiment, in particular, the lateral umklapp scattering proves to be essential. For a monolayer graphene, the question arises of how strongly the interaction with the substrate modifies the symmetry properties of the initial and the final states. Here, this question is addressed by comparing the symmetry of the emission from the monolayer and bilayer graphene. This estimate suggests that the reflection of the outgoing photoelectron from the underlying substrate may explain the experimentally observed symmetry breaking.

## 2. Computational Methodology and Approximations

According to the one-step theory of photoemission [25,26,27,28,29] the photocurrent $I(\left(k_{\left|\right|}\omega \right)$ is proportional to the probability of the transition from the initial state $|\;i\;k_{\left|\right|}\;\rangle$ to the time reversed LEED state $|\;f\;k_{\left|\right|}\;\rangle$:

(1) $$I\left(\omega ,k_{\|}\right)∼\sqrt{E_{f}-E_{\mathrm{vac}}}\;{\left|\langle \;f\;k_{\left|\right|}\;|\hat{o}|\;i\;k_{\left|\right|}\;\rangle \right|}^{2},$$

where $\langle \;r\;|\;f\;k_{\left|\right|}\;\rangle =\Phi_{\mathrm{LEED}}^{*}\left(r\right)$, and $k_{\left|\right|}$ is the crystal momentum parallel to the surface. In the dipole approximation the perturbation operator is $\hat{o}=-i\nabla \cdot e$, where e is the light polarization vector. Thereby, the dielectric response of the electronic system is neglected. In principle, the related spatially inhomogeneous exciting field may lead to sharp structures in the photon energy dependence of the photoemission intensity. Such local field effects are known to be important below the plasma frequency, where the conditions for the excitation of the multipole plasmon may be met [35]. Here, the energies well above the plasmon are considered and, although the dielectric response may be tangible also at the higher energies, the experience with other materials [36] suggests that there one can hardly expect the local fields to give rise to sharp spectral features.

**Figure 1:**
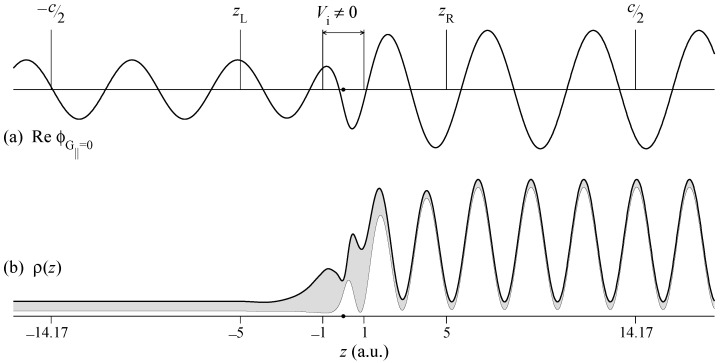
Wave function of the LEED state at $k_{\left|\right|}=1.633$ Å${}^{-1}$ along $\bar{\Gamma }\bar{K}$ and $E-E_{F}=35$ eV. (**a**) Central beam $\phi_{G_{\left|\right|}=0}\left(z\right)$ of the Laue representation (2). (**b**) Density profile ρ(z), see Equation (3). The graphene monolayer is at z=0. The shaded area in graph (**b**) shows the contribution from the $G_{\left|\right|}\neq 0$ surface Fourier harmonics.

The LEED wave function $\Phi_{\mathrm{LEED}}\left(r\right)$ is a scattering solution for a plane wave incident from vacuum with the final state energy *E*. Inside the graphene layer it satisfies the Schrödinger equation with the Hamiltonian $\hat{H}=-\Delta +V\left(r\right)-iV_{i}$. Here an imaginary potential $-iV_{i}$ is added to the crystal potential V(r) to allow for the inelastic scattering of the outgoing electron. In photoemission from semi-infinite crystals, the absorbing potential simulates the surface sensitivity of photoemission and leads to a momentum broadening perpendicular to the surface. For finite-thickness films the interaction with the electronic system is limited to a thin layer. In the present calculation it is chosen to vanish outside a thin slab between z=−1 and 1 a.u., see Figure 1. The results were found to be rather insensitive to $V_{i}$ in the range from 1 to 4 eV: The absorbing potential smoothes the electron transmission curves T(E) and constant initial state (CIS) spectra I(ω) and reduces the peak intensities of I(ω) (by around 20–30% per 1 eV increase in $V_{i}$). Otherwise $V_{i}$ does not affect the shape of the curves. Increasing $V_{i}$ from 1 to 4 eV leads to an increase by 20% of the peak value of the intensity ratio in Figure 4a. The present calculations are for $V_{i}=1$ eV.

In the electron diffraction calculation the wave is incident from the right, and the space is divided into three regions: (i) left vacuum half-space $z<z_{L}$, which contains the transmitted plane waves, (ii) scattering region $z_{L}\leq z\leq z_{R}$, and (iii) right vacuum half-space $z>z_{R}$, which contains the incident plane wave and reflected waves. In the scattering region the wave function is a linear combination of the eigenfunctions ψ of an auxiliary three-dimensional *z*-periodic crystal, which contains the scattering region as a part of the unit cell, Figure 1. The solution of the scattering problem consists in constructing a linear combinations of the basis functions ψ that satisfies the Schrödinger equation in region (ii) and at the planes $z_{L}$ and $z_{R}$ matches the function and derivative of the plane-wave representations in regions (i) and (iii), respectively. This is achieved by the variational embedding method introduced in Ref. [34]. Thus, a Laue representation of the LEED state is constructed:

(2) $$\Phi_{\mathrm{LEED}}\left(r_{\left|\right|},z\right)=\sum_{G_{\left|\right|}}\phi_{G_{\left|\right|}}\left(z\right)\exp\left[i\left(k_{\left|\right|}+G_{\left|\right|}\right)\;r_{\left|\right|}\right],$$

which in the present calculation comprises 19 surface reciprocal vectors $G_{\left|\right|}$. The lattice constant of the auxiliary crystal along *z* was c=15 Å, and the basis set in region (ii) comprised the ψ functions with energies up to about 40 eV above the highest energy of interest, which amounts to around 200 ψ functions for the monolayer graphene. The Laue representation (2) is obtained by a straightforward expansion of the all-electron wave function in terms of 11,997 plane waves (G≤11 a.u.${}^{-1}$). The potential V(r) of the auxiliary crystal is determined self-consistently within the local density approximation by the full-potential augmented Fourier components method [37].

An example of the scattering solution for $k_{\left|\right|}=1.633$ Å${}^{-1}$ along $\bar{\Gamma }\bar{K}$ and E=35 eV is presented in Figure 1. Figure 1b shows the density profile of this LEED state

(3) $$\rho \left(z\right)=\int |\;\Phi_{\mathrm{LEED}}\left(r_{\left|\right|},z\right){\;|}^{2}\;dr_{\left|\right|}$$

and demonstrates that in the interior of the graphene layer the contribution from the $G_{\left|\right|}\neq 0$ harmonics strongly exceeds the $G_{\left|\right|}=0$ contribution. It is the $G_{\left|\right|}\neq 0$ contribution that makes a single-plane-wave approximation for the final state $\Phi_{\mathrm{LEED}}^{*}$ unrealistic and misleading, see a detailed analysis in Ref. [38].

## 3. Results and Discussion

In this section, the calculation of photoemission form graphene is presented in the range ℏω=20 to 60 eV with the emphasis on the comparison with the experiments of Refs. [13,18]. The spectra are analyzed in terms of dipole transitions to the $\Phi_{\mathrm{LEED}}^{*}$ states for an all-electron Kohn–Sham potential. Detailed analysis of the monolayer and bilayer graphene is given in Section 3.1 and Section 3.2, respectively.

### 3.1. Monolayer Graphene

**Figure 2:**
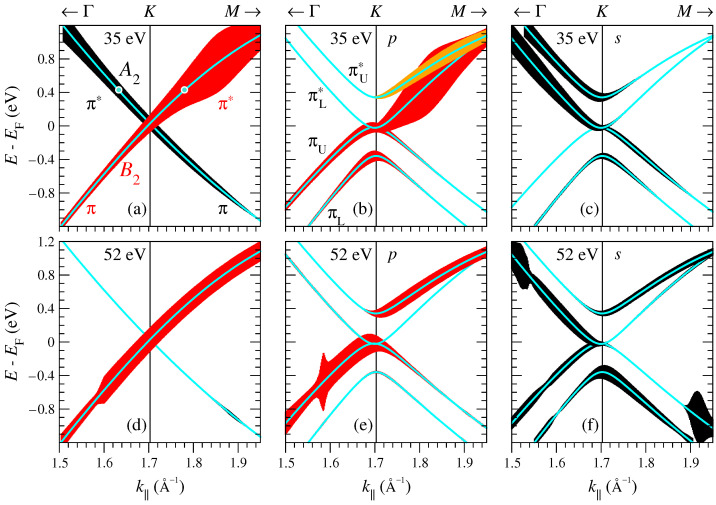
Crystal-momentum dependence of the photocurrent from graphene along $\bar{\Gamma }\bar{K}$: (**a**,**d**) monolayer; (**b**,**c**,**e**,**f**) bilayer. (**a**–**c**) ℏω=35 eV; (**d**–**f**) ℏω=52 eV. Light is incident along $\bar{\Gamma }\bar{K}$ at an angle of 18°. Intensity at *p* polarization is shown by red and at *s* polarization by black shading. The vertical extent of the shaded area is proportional to the relative intensity in the same graph (intensity is normalized differently in each of the graphs). The photon energy dependence of the intensity can be inferred from Figure 3. Because of the strict parity selection rules, for the monolayer graphene both polarizations are shown in the same graph (**a**,**d**). In graph (**a**) the two circles mark the initial states considered in Figure 4.

Calculated polarization dependence of the photoemission from the monolayer and bilayer graphene is shown in Figure 2 for $k_{\left|\right|}$ along $\bar{\Gamma }\bar{K}$ around the DP for two photon energies ℏω=35 and 52 eV. The light incidence plane intersects the surface in the $\bar{\Gamma }\bar{K}$ line, and the angle of incidence is 18°, as in the experiment of Ref. [18]. For the monolayer graphene, the two branches have different parities under the reflection in $\bar{\Gamma }\bar{K}$ so the ascending branch ($B_{2}$ symmetry) is visible only in *p* polarization and the descending branch ($A_{2}$) only in the *s* polarization. A similar trend is observed in the graphene bilayer, only here the π states are not parity eigenfunctions, so every state is visible in both polarizations, albeit with a striking intensity asymmetry. The absolute intensities and the asymmetry, however, depend on the photon energy, as seen from the comparison of Figure 2a–c,d–f.

**Figure 3:**
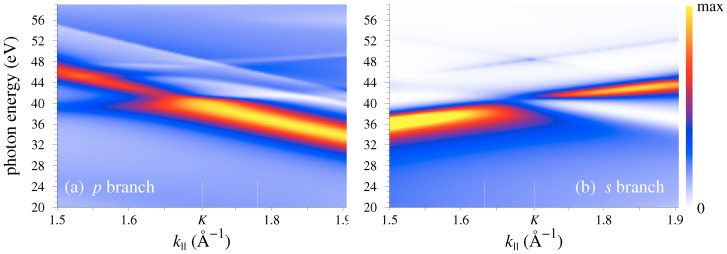
Photocurrent distribution in photon energy and crystal momentum from the π and $\pi^{*}$ states for the same setup as in Figure 2. (**a**) $B_{2}$ states (*p* branch). (**b**) $A_{2}$ states (*s* branch). In each graph the two white vertical ticks show the $\bar{K}$ point and the $k_{\left|\right|}$ point presented in detail in Figure 4.

In agreement with the experimental observation of Ref. [13], in the monolayer graphene over a wide $k_{\left|\right|}$ interval around $\bar{K}$ the intensity of the *s* branch at 52 eV is an order of magnitude lower than at 35 eV. For each of the two photon energies 35 and 52 eV the intensity changes slowly and steadily with $k_{\left|\right|}$, however, this is not the case for the ℏω interval between 35 and 52 eV, as illustrated by the intensity distribution $I(k_{\left|\right|},\omega )$ in Figure 3. The *p* branch manifests a sharp peak, which over the interval from 1.7 to 1.9 Å${}^{-1}$ disperses from 40 to 33 eV and is followed by a minimum and a set of weaker structures at higher energies. The *s* branch has two sharp maxima dispersing upwards: The one due to π states (below the DP) in the second BZ around ℏω=42 eV and the one due to $\pi^{*}$ states (above the DP) in the first BZ around 38 eV. Apart from that, the *s* branch of both π and $\pi^{*}$ states manifests a sharp nondispersive dip at around 45.5 eV: The intensity drops by a factor of 5 over an interval of about 3 eV and then rapidly rises again.

**Figure 4:**
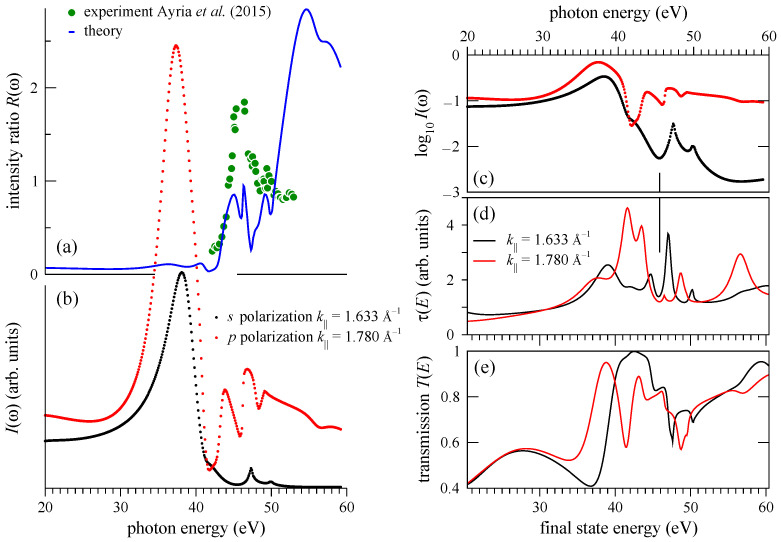
(**a**) Photon energy dependence of the relative intensity R(ω), see Equation (4), of the emission from the $B_{2}$ state ($I_{p}$) at $k_{\left|\right|}=1.780$ Å${}^{-1}$ and $A_{2}$ state ($I_{s}$) at $k_{\left|\right|}=1.633$ Å${}^{-1}$ for the polarization angle $\phi =78^{\circ }$ (4.3% of *p* and 95.7% of *s* polarization). Both initial states are located at about 0.4 eV above the DP. Full circles show the measurements of Ref. [18] (digitized from Figure 5 in that work). (**b**) Calculated constant initial state spectra $I_{s}\left(\omega \right)$ (black) and $I_{p}\left(\omega \right)$ (red). (**c**) Logarithmized intensities $\log_{10}I_{s}\left(\omega \right)$ (black) and $\log_{10}I_{p}\left(\omega \right)$ (red). (**d**) Dwell time τ(E): The probability to find the electron in the LEED state within a layer between z=−2 and 2 a.u., see Figure 1. The final state energy *E* is relative to the DP. (**e**) Electron transmission T(E) for $k_{\left|\right|}=1.633$ Å${}^{-1}$ (black) and $k_{\left|\right|}=1.780$ Å${}^{-1}$ (red). To facilitate the comparison, the *E* range in graphs (**d**) and (**e**) is shifted by 0.4 eV (initial state energy) relative to the ℏω range in graph (**c**).

The constant initial state spectra for the $\pi^{*}$ states at $k_{\left|\right|}=1.633$ and 1.780 Å${}^{-1}$ are shown in Figure 4b, and their ratio

(4) $$R\left(\omega \right)=\frac{I_{p}\left(\omega \right)\cos^{2}\;\phi }{I_{s}\left(\omega \right)\sin^{2}\;\phi },$$

where $\phi =78^{\circ }$ is the experimental polarization angle, is compared to the experiment [18] in Figure 4a. The minimum of the *s* branch at ℏω=45.5 eV gives rise to a maximum in R(ω) very close in energy to the measured maximum at 46 eV. The calculated magnitude of R(ω) is two times lower than in the experiment, which can be considered a satisfactory agreement in view of the fact that it is related to a deep minimum in the denominator, i.e., to the cancellation effects in the momentum matrix element (1) for *s* polarization. Naturally, in this situation the observables are especially sensitive to the accuracy of the wave functions, and an exact knowledge of all details is needed to achieve a perfect agreement. On the theoretical side, the discrepancy may arise from the neglect of the dielectric response (dipole approximation for the perturbation operator) and possibly from using the Kohn–Sham eigenfunctions for quasiparticles. Computational uncertainty related to the accuracy of the wave functions can hardly tangibly contribute to the discrepancy (judging by the convergence of the observables).

**Figure 5:**
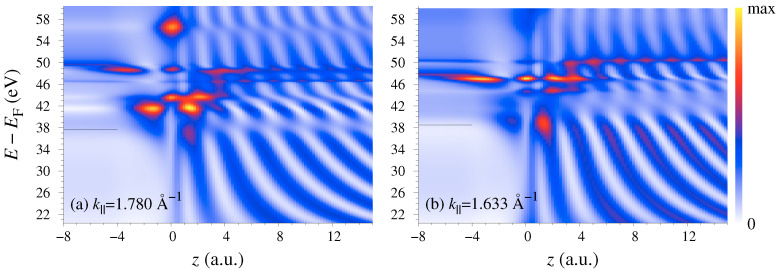
Energy dependence of the density distribution ρ(z) in the LEED states: (**a**) $k_{\left|\right|}=1.780$ Å${}^{-1}$ effects the *p* branch emission, and (**b**) $k_{\left|\right|}=1.633$ Å${}^{-1}$ the *s* branch, see Figure 4b. The horizontal bars at 37.7 eV (**a**) and 38.5 eV (**b**) indicate the final states at the intensity peaks in Figure 4b.

These results thereby establish the R(ω) peak to originate from the rapidly changing character of the final state wave function, and it is tempting to relate it to gross features of the scattered wave. In particular, because the initial states are confined to the graphene layer it is instructive to consider the spatial character of the LEED states as a function of energy, see Figure 5. The electron scattering by the graphene monolayer was first studied theoretically in Ref. [5], where the existence of scattering resonances was predicted that manifested themselves as rapid variations of the transmission probability T(E) accompanied by a sharp enhancement of the density ρ(z) at the graphene layer. Figure 5 shows that around the $\bar{K}$ point the resonances have rather complicated spatial structure, which strongly changes with $k_{\left|\right|}$. Consequently, the probability to find the scattered electron at the graphene layer—the so-called dwell time τ(E)—varies with energy. The τ(E) curves for $k_{\left|\right|}=1.633$ and 1.780 Å${}^{-1}$ are shown in Figure 4d (the probability density ρ(z) was integrated from z=−2 to 2 a.u.). Both curves show rich structure, but the τ(E) variations do not correlate with those of the photocurrent, and although the dip in the $I_{s}\left(E\right)$ curve coincides with a minimum in the τ(E) curve, the former drops much deeper than the latter. Generally, the τ(E) variations are much weaker than the variations of the photocurrent, which points to the importance of the interference between different $G_{\left|\right|}$ contributions to the photoemission matrix element also for *p* polarization.

Figure 4e shows the electron transmission spectra T(E), i.e., the ratio of the transmitted current at −∞ to the incident current from +∞. The T(E) curves show a minimum (at E=37 eV for $k_{\left|\right|}=1.633$ Å${}^{-1}$ and 34 eV for 1.780 Å${}^{-1}$) followed by a maximum (at 42.5 and 39 eV, respectively), which is a signature of the scattering resonance [5]. The photoemission intensity peaks are located at ℏω=37.3 eV for $I_{p}$ and 38.1 eV for $I_{s}$, close to the inflection points of the respective T(E) curves, E=37 and 39 eV. Although it is not surprizing that the sharp enhancement of the intensity occurs in the resonance region, it cannot be directly related to the gross features of the final state, such as the transmission probability or density distribution.

### 3.2. Bilayer Graphene: Relaxation of Parity Selection Rules

**Figure 6:**
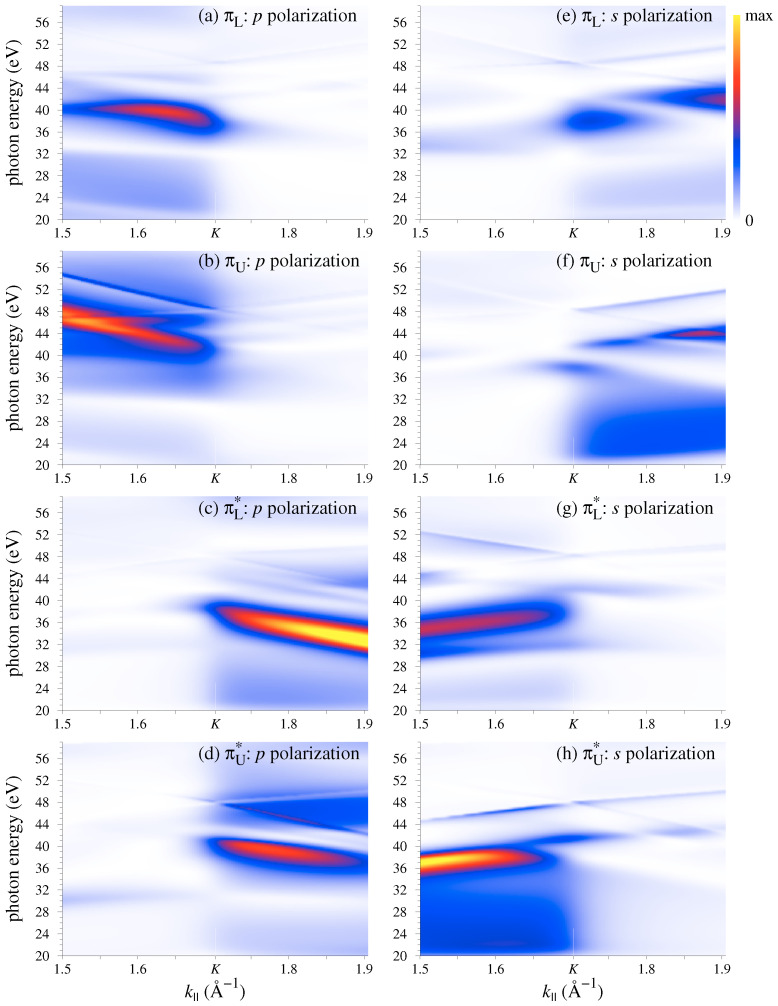
Photocurrent distribution in photon energy and crystal momentum from the π and $\pi^{*}$ states of bilayer graphene, see Figure 2b for notation. (**a**–**d**) *p* and (**e**–**h**) *s* polarization. (Horizontal cross-sections of the maps at ℏω=35 and 52 eV are presented in Figure 2b,c,e,f).

Crystal momentum-photon energy distribution of the photocurrent for both light polarizations is presented in Figure 6 for the four bands around the $\bar{K}$ point: Concave down bands $\pi_{L}$ and $\pi_{U}$ and concave up bands $\pi_{L}^{*}$ and $\pi_{U}^{*}$, see Figure 2b for notation. Similar to the monolayer graphene, the ascending branches are highlighted by the *p*-polarized light, while the descending ones by the *s*-polarized light. For both polarizations the CIS of each of the bands manifests a strong peak, which disperses downwards in ℏω with $k_{\left|\right|}$ for *p* polarization and upwards for *s* polarization, compare Figure 3 and Figure 6.

However, because the bilayer is not invariant under the reflection in the $\bar{\Gamma }\bar{K}$ line the parity selection rules are relaxed, and at certain ℏω the ascending and descending branches for a given light polarization may have comparable intensities. As seen in Figure 6, this happens when the intensity of the *p*- or *s*-highlighted branch drops off for reasons not related to the $\bar{\Gamma }\bar{K}$ reflection properties. For *p* polarization this occurs, for example, around ℏω=31 eV, where the descending $\pi_{U}^{*}$ branch turns out to have higher intensity than the ascending branch (Figure 6d). For *s* polarization one can observe such asymmetry inversion for the $\pi_{L}$ branch around 34 eV (Figure 6e).

**Figure 7:**
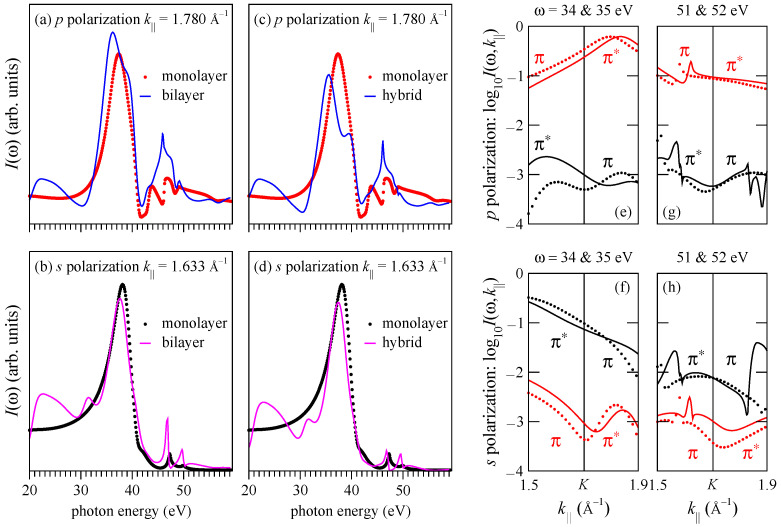
(**a**,**b**) Comparison of the CIS spectra for the monolayer (dots) and bilayer graphene (lines): (**a**) *p* polarization, (**b**) *s* polarization. The bilayer curves are a sum of the $\pi_{L}^{*}$ and $\pi_{U}^{*}$ spectra, see Figure 2b for notation. (**c**,**d**) Comparison of the monolayer (dots) and hybrid-model (lines) CIS spectra: (**c**) *p* polarization, (**d**) *s* polarization. The monolayer curves in graphs (**a**,**c**) and (**b**,**d**) are the same as the curves of the respective colors in Figure 4b. (**e**,**f**,**g**,**h**) Contrast between the $B_{2}$ and $A_{2}$ branches by the hybrid model. Crystal-momentum dependence of the intensity from the π and $\pi^{*}$ states of the $B_{2}$ (red) and $A_{2}$ (black) branch for four photon energies: (**e**,**f**) ℏω=34 eV (lines) and 35 eV (dots); (**g**,**h**) ℏω=51 eV (lines) and 52 eV (dots) for *p* polarization (**e**,**g**) and *s* polarization (**f**,**h**).

In spite of the rather strong effect of the interlayer interaction on the π states, the overall shape of the CIS curves is rather close for the monolayer and bilayer graphene, see Figure 7a,b. Let us now draw on these results to comment on the observation in Ref. [13] that in the monolayer graphene the emission from the $B_{2}$ band is visible also in the *s*-polarized light: This may be due to the scattering of the outgoing electron by the underlying substrate. It is reasonable to assume that the scattering by the substrate surface is comparable to the interlayer scattering in the bilayer graphene. To estimate its implications for the selection rules, we construct the matrix elements in Equation (1) between the initial states of the monolayer graphene (which are parity eigenfunctions) and the $\Phi_{\mathrm{LEED}}^{*}$ states of the bilayer graphene. This hybrid model yields the intensity distributions $I(k_{\left|\right|},\omega )$ very similar to those in Figure 3. As an example, the hybrid-model CIS curves for the $\pi^{*}$ states at $k_{\left|\right|}=1.780$ and 1.633 Å${}^{-1}$ are compared to the monolayer spectra in Figure 7c,d.

The extent to which the scattering by the second graphene layer relaxes the selection rules is revealed by Figure 7e–h, which compare the $k_{\left|\right|}$ dependence of the emission from the $B_{2}$ and $A_{2}$ branch for both light polarizations by the hybrid model. For *p* polarization (Figure 7e–g) the $B_{2}$ branch is about two orders of magnitude stronger than the $A_{2}$ branch. This is not surprising, as the dark corridor was also observed in photoemission from the bulk graphite [21,22]. The situation is somewhat different for *s* polarization: again, for ℏω=34 and 35 eV the $A_{2}$ branch is two orders of magnitude more intense than the $B_{2}$ branch around the $\bar{K}$ point, but below the DP the intensities of the $A_{2}$ and $B_{2}$ branches become closer to each other in moving to lower energies, i.e., away from the $\bar{K}$ point, see Figure 7f. As we have seen for the two selected $k_{\left|\right|}$, the intensity drop-off above the resonance is stronger for the $A_{2}$ branch than for the $B_{2}$ branch. Figure 7h demonstrates that this is the case over a wide $k_{\left|\right|}$ interval around the $\bar{K}$ point and that at ℏω=52 eV the two branches are much closer in intensity than at 35 eV. This qualitatively agrees with the measurements of Ref. [13], where the overall contrast between the two branches was considerably stronger for 35 eV than for 52 eV. Furthermore, Figure 7f–h show that the contrast may be very sensitive to the photon energy: A variation of ℏω by 1 eV may change the intensity by a factor of 2. The hybrid model thus shows that the scattering of the photoelectron emitted from the graphene monolayer by the substrate may be sufficiently strong to break the symmetry of the photoexcitation. Another reason for the symmetry breaking is the spin–orbit interaction, as discussed in Ref. [13]. This effect is neglected in the present calculation.

## 4. Summary and Conclusions

The present application of the one-step theory of photoemission to the monolayer and bilayer graphene demonstrates a strong effect of the in-plane scattering of the outgoing photoelectron on the photoemission intensity. The continuum spectrum of graphene contains scattering resonances first discovered in Ref. [5] and interpreted as due to the coupling of the in-plane and perpendicular motions. At the $\bar{K}$ point the resonance is located around 38 eV above the DP, and the present theory predicts the photoemission from the Dirac cone to be strongly enhanced in the resonance region both for *p* and for *s* light polarization. Above the resonance the intensity drops more strongly for *s* than for *p* polarization, in agreement with the experiment [13]. In the interval up to about 15 eV above the resonance the scattering states have very complicated and rapidly changing structure, which is reflected both in the electron transmission and in the photoemission spectra, although no obvious correlation between the T(E) and I(ω) curves is observed. (This means in particular that a single-plane-wave approximation for the final state would be completely inappropriate for graphene). The presence of this fine structure offers the possibility to relate the theoretically predicted spectral features to the measured ones and to verify the validity of the approximations involved, in particular, how accurately the density-functional derived potential simulates the excited states (it is known to underestimate the quasiparticle energies). The good agreement of the calculated energy dependence of the $I_{p}/I_{s}$ ratio (4) with the experiment [18] suggests that the self-energy shift is quite moderate (around 0.5 to 1 eV), as expected from previous experience [6,30,31,32,33], and that the Kohn–Sham quasiparticles are a good approximation for graphene.

The comparison of the monolayer and bilayer spectra is instructive in order to estimate the effect of the scattering by the substrate on the symmetry breaking in photoemission from the monolayer graphene. The true structure of the interface between the graphene monolayer and the substrate is very difficult to include in an ab initio calculation because the mismatch between the lattices of the substrate and graphene as well as the presence of the reconstructed buffer layer would require a huge supercell. Instead, we resorted to a hybrid model that combines the initial states of the monolayer graphene (which have $B_{2}$ or $A_{2}$ symmetry) with the final states of the bilayer (which are not symmetry eigenfunctions). Such a heuristic model is justified in view of the close similarity of the gross features of the monolayer and bilayer spectra. It shows that the relaxation of the selection rules is most important in the region of low intensity (above ℏω=50 eV for *s* polarization) and that the symmetry breaking observed in Ref. [13] can be explained by the scattering from the substrate. Generally, at low intensities, the emission is very sensitive to this effect, which should be kept in mind in theoretically modeling this energy range with ideal free-standing graphene.

## Data Availability

Data is contained within the article.
